# Population with an increased risk of severe COVID-19 in Germany. Analyses from GEDA 2019/2020-EHIS

**DOI:** 10.25646/7859

**Published:** 2021-04-21

**Authors:** Alexander Rommel, Elena von der Lippe, Marina Treskova-Schwarzbach, Stefan Scholz

**Affiliations:** 1 Robert Koch Institute, Berlin Department of Epidemiology and Health Monitoring; 2 Robert Koch Institute, Berlin Department of Infectious Disease Epidemiology

**Keywords:** SARS-COV-2, COVID-19, PRE-EXISTING CONDITIONS, SECONDARY DISEASES, RISK FACTORS, HOSPITALISATION, MORTALITY

## Abstract

Only a minority of people who test positive for COVID-19 develop a severe or critical form of the disease. Many of these have risk factors such as old age or pre-existing conditions and, therefore, are at the focus of protective measures. This article determines the number of people at risk in Germany and differentiates them according to age, sex, education, household type and federal state. The analyses presented here are based on data from the German Health Update (GEDA) 2019/2020-EHIS, which was carried out as a nationwide cross-sectional telephone-based survey between April 2019 and October 2020. The definition of being at increased risk of severe COVID-19 is primarily based on a respondent’s age and the presence of pre-existing conditions. Around 36.5 million people in Germany are at an increased risk of developing severe COVID-19. Of these, 21.6 million belong to the high-risk group. An above-average number of people at risk live alone. The prevalence of an increased risk is higher among middle-aged men than among women of the same age, and significantly higher among people with a low level of education than among people with a high level of education. The highest proportion of people with an increased risk live in Saarland and in the eastern German federal states. When fighting the pandemic, it is important to account for the fact that more than half of the population aged 15 or over is at increased risk of severe illness. Moreover, the regional differences in risk burden should be taken into account when planning interventions.

## 1. Introduction

SARS-CoV-2 (Severe Acute Respiratory Syndrome Coronavirus 2) and the Coronavirus Disease 2019 (COVID-19), have had a strong political and social impact on life in Germany in 2020. After a first wave of infections in spring and a sharp decline in new cases in summer, the numbers began to rise again significantly in October [1]. By the second half of November, the number of deaths clearly exceeded the level in spring [2]. The disease burden from death due to COVID-19 in 2020 is above the level that would normally be expected for lower respiratory infections during this period. Men account for almost two thirds of years of life lost to COVID-19, and people aged 70 for a share of more than 70% [2].

The vaccines that have been gradually approved since the end of 2020 are key to avoiding infections and severe illness. Since the number of vaccines available will be limited for the foreseeable future, access to vaccines needs to be regulated [3]. This is particularly important if vulnerable groups are to be protected. The priority groups for COVID-19 vaccinations broadly fall into two categories: in addition to people in occupations deemed essential (e.g. nursing staff), who are often at high risk of infecting themselves and others, the focus is primarily placed on people with an increased risk of developing a severe form of the disease (vulnerable groups).

**GEDA 2019/2020-EHIS**

Fifth follow-up survey of the German Health Update

**Data holder:** Robert Koch Institute

**Objectives:** Provision of reliable information on the health status, health behaviour and health care of the population living in Germany, with the possibility of European comparisons

**Study design:** Cross-sectional telephone survey

**Population:** German-speaking population aged 15 and older living in private households that can be reached via landline or mobile phone

**Sampling:** Random sample of landline and mobile telephone numbers (dual-frame method) from the ADM sampling system (Arbeitskreis Deutscher Markt- und Sozialforschungsinstitute e.V.)

**Sample size:** 23,001 respondents

**Study period:** April 2019 to September 2020

**GEDA survey waves:**

- GEDA 2009
- GEDA 2010
- GEDA 2012
- GEDA 2014/2015-EHIS
- GEDA 2019/2020-EHIS

**Further information in German is available at**

www.geda-studie.de

Data from the first COVID-19 wave demonstrates that only a minority of people who had a COVID-19 infection developed severe illness. While around 80% of the people who tested positive developed mild cases, the rest suffered from complications such as pneumonia, and required inpatient or intensive care, or died. These are classified as severe or critical forms of the disease [4]. In order to reduce the burden of disease caused by COVID-19, therefore, it makes sense to prioritise people for the vaccine who are more likely to be affected by severe illness. The majority of these people have already reached an advanced age or have certain pre-existing conditions [4]. This means that the age distribution of people with an increased risk is particularly relevant when planning vaccination programmes.

The existing evidence can be used to clearly define risk factors associated with severe COVID-19 [3]. The data demonstrate that old age is the main risk factor for hospitalisation and death, regardless of any pre-existing conditions. This evidence led the Standing Committee on Vaccination (STIKO) to recommend that older and very old people be prioritised for COVID-19 vaccination [3]. Moreover, many older people have pre-existing conditions or other risk factors. Of these, only a few conditions, such as diabetes mellitus, organ transplants, heart failure, dementia, chronic kidney disease, Down’s syndrome or severe obesity (body mass index >40), are also associated with a strongly increased risk of hospitalisation or death [3]. Several others, such as psychiatric disorders, cardiac arrhythmias, cardiovascular diseases, stroke, cancer, asthma and chronic obstructive pulmonary disease (COPD) are linked to a moderately increased risk.

This article uses current data to identify the total number of people at increased risk of developing severe COVID-19 in Germany. In addition, it develops an approach with which to implement more in-depth analyses. Based on data from the German Health Update (GEDA 2019/2020-EHIS), people aged 15 or above who have an increased risk of developing severe COVID-19 are further differentiated by age, sex, level of education, household type and federal state. Whereas the nationwide distribution of people with an increased risk can provide important information when allocating vaccines, sociodemographic factors are also useful in terms of the likelihood of gaining access to particular population groups and on how best to design information campaigns.

## 2. Methodology

### 2.1 Study design, sample and weighting

GEDA 2019/2020-EHIS is a nationwide cross-sectional telephone-based survey of the resident population in Germany aged 15 or above. The study collects data using a specially designed, fully structured computer assisted telephone interview (CATI). The GEDA study has been carried out, at intervals of several years, by the Robert Koch Institute (RKI) on behalf of the German Federal Ministry of Health since 2008, and is part of the health monitoring system at the RKI [5, 6]. The European Health Interview Survey (EHIS) was supplemented and fully integrated into the study [7]. The current GEDA wave is based on a random sample of landline and mobile phone numbers derived from a telephone sample collected by ADM (Arbeitskreis Deutscher Markt- und Sozialforschungsinstitute e.V.) [8, 9]. The sample covers the population aged 15 or above living in private households and residing in Germany at the time of data collection [8, 10].

The interviews were undertaken between April 2019 and October 2020 by an external market and social research institute under the continuous supervision of the RKI. A total of 23,001 (12,111 female, 10,890 male) people who took part in the GEDA 2019/2020-EHIS study provided complete interviews. The response rate was calculated using the standards drawn up by the American Association for Public Opinion Research (AAPOR) and amounted to 21.6% (RR3) [11]. Design weighting was initially carried out for the different selection probabilities (mobile and landline networks) using a standard calculation method as part of the dual-frame design. The sample was then adjusted to ensure that it reflected official population figures in terms of age, sex, federal state, district type (as of 31 December 2019) and education (in line with the pattern identified in the 2017 microcensus using the International Standard Classification of Education – ISCED classification) [8, 10].

### 2.2 Indicators

#### Pre-existing conditions

Data on the 12-month prevalence of pre-existing conditions was gathered using the question: ‘Have you had any of the following illnesses or complaints in the last 12 months?’ (For a list of the conditions concerned, see Chapter 2.3).

#### Body weight and body mass index

Body weight and height are based on information provided by the respondents. Data on height was collected using the question: ‘How tall are you if you are not wearing shoes?’ and was recorded in centimetres. Data on body weight was collected with the question: ‘How much do you weigh if you are not wearing clothes or shoes? Please enter your body weight in kilogrammes’. Body Mass Index (BMI) is calculated using the ratio of body weight to body height squared (kg/m^2^).

#### Need for help

People aged 55 or above were asked whether they needed help with activities of daily living (ADL) [12] or with instrumental activities of daily living (iADL) [13]. ADLs include eating and drinking, getting up from or sitting down on a bed or chair, dressing/undressing, using the toilet, or bathing and showering. iADLs include meal preparation, making phone calls, shopping, taking medication, light or occasionally heavy housework, and administrative tasks. Respondents who said they needed help with at least two of these activities were categorised as needing help.

#### Social determinants

Education levels are based on the CASMIN system (Comparative Analyses of Social Mobility in Industrial Nations) and used as an indicator of social status. Academic and vocational qualifications are used to distinguish between three groups: low, medium and high levels of education [14].

The respondents are also categorised by household type: people living alone are distinguished from couples without children, families with children and people living together in a different or unknown household type. Families also include single parents living with adult children in the household. Other household types include individuals living with people who are not partners or family members.

### 2.3 Definition of risk

Two types of risk groups are defined: the first includes everyone who is at increased risk of developing severe COVID-19; the second includes everyone within this group who has a strongly increased risk of developing severe disease. The definition of the two risk groups was based on findings derived from a systematic literature analysis carried out by the RKI as an umbrella review. The analysis was undertaken as part of the process used by the Standing Committee on Vaccination at the RKI to draw up its recommendations on vaccination priority [3, 15]. Only those conditions or risk factors could be taken into account by the applied risk definition, that were actually surveyed in GEDA 2019/2020-EHIS. In order to compensate to some extent for unmeasured morbidity, people in need of additional help are also included in the definition. The risk groups were defined using the criteria set out in Table 1.

### 2.4 Sensitivity analysis

A sensitivity analysis was undertaken with data from the previous GEDA 2014/2015-EHIS study [16]. This allowed to widen the definition by including pre-existing conditions that had been examined in 2014/2015 but not in 2019/2020. An analysis was undertaken using heart failure and cancer as examples to determine how the size of the risk group in Germany might change if people with these conditions were included in the group considered to be at risk.

The diseases were selected because they were associated with a relative risk >1 of hospitalisation (heart failure) or death (heart failure, cancer) according to literature analysis [3].

### 2.5 Statistical analysis

Weighted proportions and extrapolated population numbers for people with an increased or strongly increased risk for severe COVID-19 are depicted by age, sex, education, household type and federal state. A statistically significant difference between subpopulations is assumed where p-values are less than 0.05 (using a Pearson test for survey samples). All analyses were performed using StataSE 15.1 (Stata Corp., College Station, TX, USA, 2017).

## 3. Results

### 3.1 Groups at risk of developing severe COVID-19

The results show that 51.9% of the population in Germany aged 15 or over is at increased risk of developing severe COVID-19. Extrapolated to the German population, this corresponds to about 36.5 million people. 51.1% of these individuals are women and 48.9% are men. 30.6% of the population aged 15 or over has a strongly increased risk of developing severe COVID-19. This corresponds to 21.6 million individuals being at high risk in Germany. Of these, 53.7% are women and 48.3% are men.

**Figure 1:**
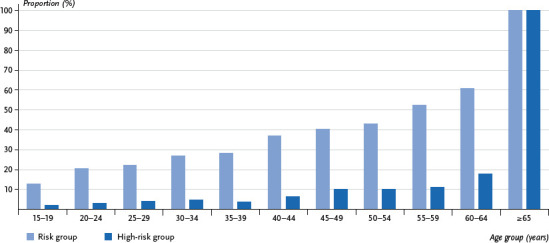
Population with an increased risk of severe COVID-19; share of risk group including the high-risk group by age (n=11,880 women, n=10,816 men) Source: GEDA 2019/2020-EHIS

The risk of developing severe COVID-19 increases steadily with age, beginning at a young age. The proportion of people at increased risk is 20.5% of 20- to 24-year-olds, 40.2% of 45- to 49-year-olds and 60.9% of 60- to 64-year-olds. In contrast, the proportion of people in the high-risk group initially remains at a low level among younger and middle-aged people. Up to the fifth decade of life, less than one-in-ten people belong to the high-risk group. In fact, only 17.7% of 60- to 64-year-olds are at high risk. Therefore, most people are at a high risk due to their advanced age (Figure 1). Nonetheless, many middle-aged people also are at risk of developing severe COVID-19. 15.5 million people in Germany under the age of 60 have an increased risk and 3.0 million have a strongly increased risk of developing severe COVID-19 (Annex Table 1).

The data demonstrate a statistically significantly higher proportion of men in the group with an increased risk of severe COVID-19. This particularly applies to middle-aged men. The difference between men and women is most pronounced between the ages of 45 and 49, with 35.3% of women and 45.0% of men in this age group deemed to be at an increased risk.

### 3.2 Risk groups by education and household type

**Figure 2:**
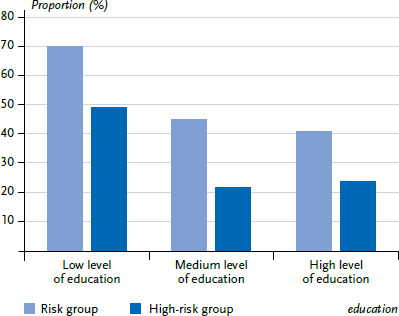
Population at increased risk of severe COVID-19; share of risk group including the high-risk group by education (n=11,880 women, n=10,816 men) Source: GEDA 2019/2020-EHIS

Clear differences were identified by education level. Whereas 69.8% of people with a low level of education in Germany belong to the risk group, this applies to 45.1% of people with a medium and 40.9% of people with a high level of education. At 49.2%, the proportion of people in the high-risk group is more than 25 percentage points higher among people with a low level of education than among people with a medium (21.9%) or high level of education (23.9%) (Figure 2).

The respondents’ household type varies significantly by risk status. Less than one-third of people who are not categorised as at risk live alone, with almost half of these individuals living with a partner or with other family members. The proportion of people living alone rises considerably with increasing risk: 45.9% of people from the risk group live alone; and 53.4% of the high-risk group does so. In total, around 16.8 million people who live alone have an increased risk of developing severe COVID-19. Nevertheless, the proportion of the risk group living with other family members is still 17.7%. As such, around 5.7 million people with an increased risk of developing severe COVID-19 are potentially being exposed to an even higher risk of infection due to the greater level of contact that they have with other people in multi-generational households (Table 2).

### 3.3 Regional differences in the risk of developing severe COVID-19

**Figure 3:**
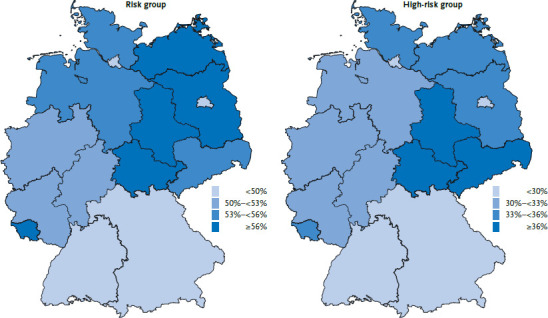
Population groups in Germany at risk of severe COVID-19 by federal state (n=11,880 women, n=10,816 men) Source: GEDA 2019/2020-EHIS

The analysis by federal state demonstrates that the population at risk is distributed unequally throughout Germany. The proportion of people in the high-risk group is highest in Saxony-Anhalt. The high-risk group is proportionally most strongly represented in Saxony and Thuringia. The share is lowest in Bavaria, Baden-Württemberg and the city states of Berlin and Hamburg. Generally, the proportions are higher in the eastern federal states than in the western federal states and are particularly low in southern Germany (Figure 3, Annex Table 2). These differences in prevalence between federal states also mean that the numbers of people at increased risk slightly differ from the figures that would be expected due to the size of the population. That is, based on the proportion of the German population aged 15 and older living in Saxony-Anhalt (2.7%), 0.98 million people would be expected to be at an increased risk of developing severe COVID-19. However, the estimates calculated using data from the GEDA 2019/2020-EHIS study demonstrate that around 1.17 million people are at risk in Saxony-Anhalt. Similarly, with 15.8% of the German population living in Bavaria, 5.74 million people would be expected to be at increased risk; the analysis found the figure to be closer to 5.46 million (Annex Table 2).

### 3.4 Sensitivity analysis

If in GEDA 2014/2015-EHIS the same definition of increased risk of developing severe COVID-19 is applied plus pre-existing conditions such as heart failure and cancer, the estimated population at risk would increase by around 465,000 people. As such, if the analyses presented here have underestimated the size of the risk group, it will have done so by probably less than about one million people

## 4. Discussion

In Germany, 36.5 million people – more than half of the population aged 15 or above – are at risk of developing severe COVID-19. Almost one-third – 21.6 million people – is at a strongly increased risk. Only a few attempts have previously been made to quantify the size of these risk groups in Germany. An extrapolation based on claims data from people with AOK health insurance found that 21.6 million people were at risk – albeit using a different definition [17]. The Central Institute for Statutory Health Insurance in Germany (Zi) identified a significantly lower prevalence among people under 60 than the results presented here [18]. However, neither of these studies included obesity as an important risk factor. Furthermore, old age is now accepted as the main risk factor for developing severe COVID-19 [3], and it was not included in these estimates as a population-related parameter. As such, the higher number of people identified as at risk in Germany by this study is plausible. Moreover, a European comparison found that Germany had one of the highest population-based risks for a severe form of COVID-19 [19].

The fact that slightly more women than men appear to be at risk can be attributed to their higher life expectancy. The prevalence of an increased risk of developing severe COVID-19 is actually higher among middle-aged men than among women of the same age. These differences between women and men, among other factors, are thought to explain the higher level of COVID-19 mortality among men [20]. Men become seriously ill with COVID-19 more frequently at a younger age and are more likely to die than women before their sixth decade of life [21, 22]. As such, the disease burden, calculated in years of life lost, is significantly higher among men than women [2].

The differences in the distribution of increased risk of developing severe COVID-19 by education also reflect the literature on socioeconomic health inequalities in Germany [23, 24]. In the case of cumulative risks, as in the present definition, such inequalities are particularly pronounced. The international literature also demonstrates that people with a low socioeconomic status are at higher risk of severe COVID-19 [25]. Regional differences in health are also well documented for Germany and show, for example, similar patterns for diabetes, high blood pressure, asthma and COPD, with higher prevalence rates in the eastern German federal states and Saarland [26–29].

Telephone-based surveys are often associated with the limitation of lower response rates compared with face-to-face surveys. However, this does not necessarily lead to a higher level of non-response bias [30]. In addition, sample weighting compensates for differences between the sample and the general population, and, therefore, for the associated biases to some extent. The results presented here assume that the measures put in place to contain the COVID-19 pandemic during the study period did not lead to any form of systematic bias. However, a temporary change in the willingness of individual population groups to participate in telephone-based surveys cannot be completely ruled out, even though initial analyses that have compared 2019 and 2020 suggest that this is not the case [8, 10]. Some important pre-existing conditions that are associated with a risk of developing severe COVID-19, such as heart failure, dementia or cancer, could not be included in the definition used in this article. However, the sensitivity analyses demonstrated that this limitation will have had little impact on the size of the risk groups.

The analyses presented here, therefore, are plausible. They describe the population in need of special protection, which should be addressed with targeted measures as a priority, if necessary. In contrast to previous analyses based on claims data, GEDA 2019/2020-EHIS also enables social determinants of an increased risk to be clearly identified. Future research should involve in-depth analyses that focus more closely on combinations of social risk factors, since sex, age, education and household composition do not independently determine a person’s risk of developing severe COVID-19. It is clear, however, that the risk of developing severe COVID-19 is distributed unequally across society. A lower level of education is associated not only with a higher risk of developing severe COVID-19, but also with a more frequent tendency to be sceptical about SARS-CoV-2 vaccinations, as a survey by the German Institute for Economic Research shows [31]. Target group-specific approaches to contain the COVID-19 pandemic could therefore be suitable for tailoring COVID-19 interventions more accurately. Furthermore, the differences in the disease burden of COVID-19 by sex indicate that men with an increased risk even at a younger age also constitute a special target group and should be particularly encouraged to take up vaccination. The different risk burden found in different regions could also be relevant when allocating vaccine. It is also important to bear in mind that 16.8 million people at risk live alone. Among these individuals are likely to be many of the 3.3 million people in need of long-term care [32] and in general many elderly community-dwelling people. As such, a proportion of these people could possibly be reached more successfully using outreach measures than opting for invitation-based procedures.

## Key statements

- Older age and certain pre-existing conditions increase a person’s risk of severe COVID-19.
- About 36.5 million people in Germany have an increased risk of severe COVID-19; 21.6 million of these belong to the high-risk group.
- 70% of people with a low level of education and 40% of people with a high level of education are at an increased risk of severe COVID-19.
- 45.9% of people in the risk group and 53.4% of people in the high-risk group live alone.
- The largest proportion of people with a high risk of severe COVID-19 live in eastern Germany and Saarland, both of which are rather sparsely populated areas.

## Figures and Tables

**Table 1:** Definition of an increased or strongly increased risk of severe COVID-19 based on data from GEDA 2019/2020-EHIS Source: Own table

**An increased risk (risk group) was assumed for individuals:**
► aged 65 or above
**or**
► with pre-existing conditions or risk factors^1^ that the literature analysis showed to be associated with a: relative risk >1 of hospitalisation [a] or death [b] [3] High blood pressure [a] Coronary heart disease/angina pectoris [b]^2^ Heart attack or chronic complications [b]^2^ Stroke or chronic complications [a]^3^ Diabetes mellitus [a, b] Bronchial asthma [a] Chronic bronchitis [a, b]^4^ Liver cirrhosis [a, b]^5^ Chronic kidney problems [a, b] Obesity (Body Mass Index ≥ 30) [a, b]
**or**
► with an additional need for help
**People were defined as belonging to a subgroup with a strongly increased risk (high-risk group) if they were:**
► aged 65 or above
**or**
► had at least one of the pre-existing conditions or risk factors that the literature analysis showed to be associated with a: relative risk >2 of hospitalisation or death [3]: diabetes mellitus, chronic kidney problems, obesity (BMI ≥ 40)^6^

BMI=Body Mass Index

^1^ The risk factors of cancer, dementia, rheumatological disease, organ transplantation, autoimmune disease, a compromised immune system and HIV infection, which were determined from the literature analysis, were not considered in GEDA 2019/2020-EHIS.

^2^ Coronary artery disease was examined as a risk factor in the underlying literature study.

^3^ Cerebrovascular disease or apoplexy was investigated as a risk factor in the underlying literature study.

^4^ Chronic obstructive pulmonary disease (COPD) was examined as a risk factor in the underlying literature study.

^5^ Chronic liver disease was investigated as a risk factor in the underlying literature study.

^6^ BMI is associated with a continuous increase in risk. The literature review found that people with a BMI ≥ 40 should be placed in the high-risk group (result not shown in [3]).

**Table 2:** Population in Germany by household type and risk of developing severe COVID-19 (n=11,880 women, n=10,816 men) Source: GEDA 2019/2020-EHIS

	No increased risk		Risk group		High-risk group	
	Number (millions)	Proportion (%)	Number (millions)	Proportion (%)	Number (millions)	Proportion (%)
Living alone	10.3	30.3	16.8	45.9	11.5	53.5
Couple without children	6.2	18.3	11.2	30.6	7.5	34.8
Family with children (including adult children)	9.9	29.1	5.7	17.7	1.6	7.3
Different household type/unknown	7.6	22.3	2.8	7.8	1.0	4.5
**Total**	**33.0**	**100**	**36.5**	**100**	**21.6**	**100**

## Appendix

**Annex Table 1:** Proportion and extrapolated number of people with an increased and high risk of severe COVID-19 by age (n=11,880 women, n=10,816 men) Source: GEDA 2019/2020-EHIS

	Risk group				High-risk group			
Age group 15 to …	%	95% CI	Number (millions)	95% CI	%	95% CI	Number (millions)	95% CI
<50 years <60 years <70 years	27.1 32.9 41.2	25.7–28.4 31.7–34.1 40.2–42.3	9.1 15.5 23.8	8.6–9.7 14.8–16.1 23.1–24.5	4.8 6.4 15.3	4.1–5.6 5.8–7.1 14.5 –16.0	1.6 3.0 8.8	1.4–1.9 2.7–3.3 8.4–9.2
**Total (≥15 years)**	**51.9**	**50.9-52.9**	**36.5**	**35.7-37.4**	**30.6**	**29.7-31.5**	**21.6**	**20.9-22.2**

CI=confidence interval

**Annex Table 2:** Proportion and extrapolated number of people with an increased and high risk of severe COVID-19 by federal state (n=11,880 women, n=10,816 men) Source: GEDA 2019/2020-EHIS

Risk group					High-risk group			
Federal state	%	95% CI	Number (millions)	95% CI	%	95% CI	Number (millions)	95% CI
Baden-Württemberg	49.0	46.3–51.7	4.61	4.27–4.95	28.4	26.2–30.9	2.68	2.43–2.93
Bavaria	49.0	46.5–51.5	5.46	5.07–5.85	28.4	26.2–30.8	3.17	2.86–3.47
Berlin	48.8	45.6–52.1	1.52	1.39–1.65	28.4	25.8–31.1	0.88	0.79–0.97
Brandenburg	59.3	54.1–64.2	1.27	1.10–1.44	34.6	30.0–39.5	0.74	0.62–0.86
Bremen	54.1	42.1–65.6	0.30	0.21–0.39	31.7	22.4–42.8	0.18	0.11–0.24
Hamburg	49.7	44.0–55.3	0.76	0.64–0.88	28.7	23.9–34.0	0.44	0.35–0.53
Hessen	50.9	47.2–54.6	2.70	2.44–2.96	30.3	27.2–33.5	1.60	1.41–1.79
Mecklenburg-Western Pomerania	59.8	52.8–66.5	0.81	0.66–0.96	35.3	29.2–41.9	0.48	0.38–0.58
Lower Saxony	53.1	49.8–56.4	3.58	3.26–3.90	32.2	29.3–35.4	2.18	1.93–2.42
Nordrhein-Westfalen	52.2	50.0–54.5	7.84	7.38–8.30	30.0	28.1–32.0	4.51	4.17– 4.85
Rhineland-Palatinate	51.7	47.3–56.2	1.80	1.59–2.02	31.0	27.2–35.0	1.08	0.93–1.24
Saarland	58.5	55.0–61.9	0.50	0.46–0.54	35.2	32.2–38.3	0.30	0.27–0.33
Saxony	53.8	49.2–58.4	1.85	1.62–2.07	37.0	32.8–41.4	1.27	1.09–1.45
Saxony-Anhalt	62.3	56.2–67.9	1.17	1.00–1.35	36.7	31.2–42.4	0.69	0.56–0.82
Schleswig-Holstein	55.1	50.3–59.8	1.37	1.19–1.55	33.5	29.4–38.0	0.83	0.71–0.96
Thuringia	59.4	53.2–65.4	1.09	0.91–1.26	37.0	31.2–43.2	0.68	0.54–0.82

CI=confidence interval
